# Target-Guided Isolation of Three Main Antioxidants from *Mahonia bealei* (Fort.) Carr. Leaves Using HSCCC

**DOI:** 10.3390/molecules24101907

**Published:** 2019-05-17

**Authors:** Weicheng Hu, Jing Zhou, Ting Shen, Xinfeng Wang

**Affiliations:** Jiangsu Collaborative Innovation Center of Regional Modern Agriculture & Environmental protection/Jiangsu Key Laboratory for Eco-Agricultural Biotechnology around Hongze Lake, Huaiyin Normal University, Huaian 223300, China; hu_weicheng@163.com (W.H.); zhou_scu@foxmail.com (J.Z.)

**Keywords:** *Mahonia bealei* (Fort.) Carr., DPPH–HPLC, HSCCC, antioxidant activity

## Abstract

*Mahonia bealei* (Fort.) Carr. is an economically important plant that is widely cultivated in Southwest China. Its leaves are commonly used for tea and contain an abundance of antioxidant compounds. However, methods of the systematic purification of antioxidants from *M. bealei* are lacking. In this study, antioxidants from this plant were effectively and rapidly enriched. First, antioxidants were screened using online 1,1-diphenyl-2-picryl-hydrazyl radical (DPPH)–high performance liquid chromatography (HPLC), followed by separation using high-speed countercurrent chromatography with an optical solvent system composed of *n*-hexane/ethyl acetate/methanol/water (1:5:1:5, v/v/v/v). Three phenolics—chlorogenic acid (**1**, 8.3 mg), quercetin-3-*O*-β-d-glucopyranoside (**2**, 20.5 mg), and isorhamnetin-3-*O*-β-d-glucopyranoside (**3**, 28.4 mg)—were obtained from the ethyl acetate-soluble fraction (240 mg) by one-step separation. The chemical structures of the phenolics were characterized by MS and NMR techniques, and the purity of each compound was >92.0% as determined by HPLC. The isolated compounds were assessed by scavenging activities on DPPH and superoxide radicals as well as cytoprotective assays, all of which showed similar trends regarding the antioxidant capacities of the compounds. Moreover, compounds **1**–**3** significantly attenuated the lipid peroxidation and antioxidant enzyme activities in H_2_O_2_-treated RAW264.7 cells. Our study demonstrated the efficiency of a newly developed integrative system for the comprehensive characterization of pure compounds from *M. bealei*, which will allow their use as reference substances.

## 1. Introduction

Oxidation is an essential biological process that participates in energy production in living organisms [1]. However, the consequences of excessive cellular levels of free radicals are relevant to the oxidation of biomolecules, leading to tissue damage, cell death, or degenerative processes including aging, cancer, cardiovascular diseases, heart diseases, and inflammation [2,3]. To reduce oxidative damage exogenously, many synthetic antioxidants have been developed, but their use is highly restricted because of their potential health hazards, including liver damage and carcinogenesis [4,5]. Thus, the effective exploitation and applications of natural antioxidants are of current interest [6,7].

Kudingcha tea is traditionally used in China and southeastern Asia, where its antioxidative capacity is well recognized [8]. *Mahonia bealei* (Fort.) Carr is a member of the Berberidanceae family and is widely distributed in the mountainous areas of southern China. It is included in the Chinese Pharmacopoeia as a Chinese folk medicine for the treatment of dysentery, jaundice, periodontitis, and bloody urine [9,10]. Its leaves, which in China are consumed traditionally as a bitter tea, contain antioxidant, anti-proliferation, anti-inflammatory, anti-bacterial, and anti-influenza activities [11,12]. However, the pharmacological testing of the leaves has been conducted mainly on extracts of the plant, such that its chemical constituents and their pharmacological activities have yet to be investigated. The present study is a detailed, target-guided chemical investigation of *M. bealei* leaves.

**Figure 1:**
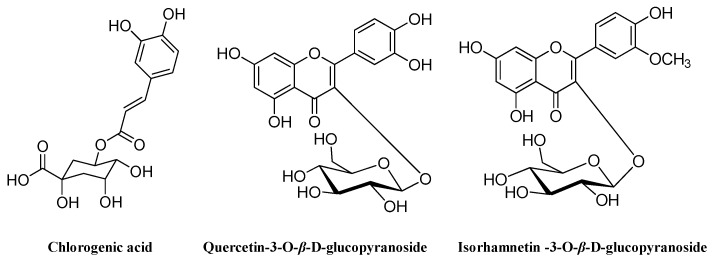
Chemical structures of three compounds obtained from *Mahonia bealei* (MBE) leaves using high-speed countercurrent chromatography (HSCCC).

To our knowledge, this is the first published report on the separation and purification of phenolic antioxidants from *M. bealei* (MBE) leaves using high-speed countercurrent chromatography (HSCCC). As part of our ongoing investigation of antioxidants in natural products, we established an efficient and simple method of rapidly preparing antioxidants from *M. bealei* leaves using online 1,1-diphenyl-2-picryl-hydrazyl radical (DPPH)–high performance liquid chromatography (HPLC) coupled with HSCCC. Chlorogenic acid (**1**), quercetin-3-*O*-β-d-glucopyranoside (**2**), and isorhamnetin-3-*O*-β-d-glucopyranoside (**3**) were obtained (Figure 1). The presence of the latter two compounds in this plant is reported here for the first time. In addition, the antioxidant capacity of the isolated compounds was evaluated in an initial report on the systematic isolation and evaluation of antioxidants in *M. bealei* leaves. The analytical method described within will facilitate the development of pure compounds from this plant to serve as reference substances.

## 2. Results and Discussion

### 2.1. Screening of Antioxidants by DPPH–HPLC

The ethyl acetate fraction of the *M. bealei* leaves showed a potent capacity to scavenge DPPH radicals, with an IC_50_ of 32.95 µg/mL (data not shown). Thus, successive DPPH–HPLC and HSCCC experiments were carried out using this fraction to screen and isolate antioxidants.

**Figure 2:**
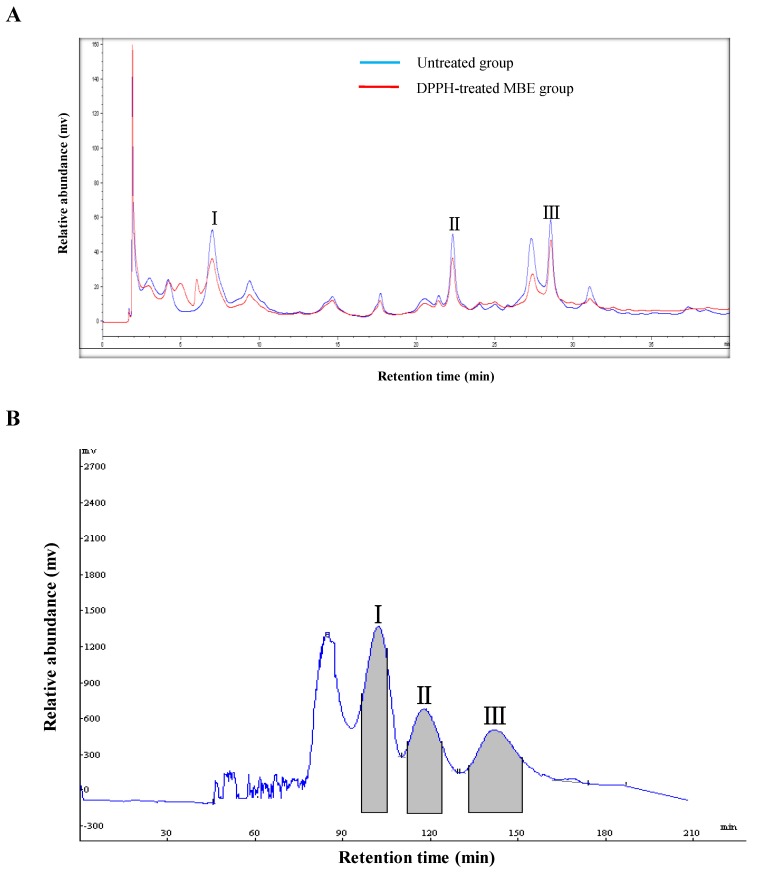
High-performance liquid chromatography (HPLC)–UV and 1,1-diphenyl-2-picryl-hydrazyl radical (DPPH)–HPLC–UV of an ethyl acetate fraction of *M. bealei* leaf extract (MBE) (**A**). HSCCC chromatogram of MBE using the *n*-hexane/ethyl acetate/methanol/water (1:5:1:5, v/v) solvent system (**B**).

The DPPH–HPLC method enables the rapid screening of antioxidants from complex mixtures, particularly natural products, with minimum sample preparation [13]. After the compounds of interest are reacted with DPPH, their peak areas in the HPLC chromatogram reduce or disappear if they contain antioxidant activity, whereas the peak areas of compounds without antioxidant activity remain essentially unchanged [14]. Untreated and DPPH-treated ethyl acetate fraction of *M. bealei* leaf extract (MBE) was processed according to the optimized separation conditions described above and then analyzed by HPLC. A comparison of the HPLC chromatograms of the untreated and DPPH-treated samples indicated three peaks (1, 2, and 3) with retention times of 6.99, 22.32, and 28.55 min, respectively. The areas of the three peaks were smaller in the samples spiked with DPPH (Figure 2A), indicating that all three compounds are antioxidants. Then, HSCCC was used to isolate and purify these active compounds.

### 2.2. HSCCC Separation and Peak Fraction Analysis

**Figure 3:**
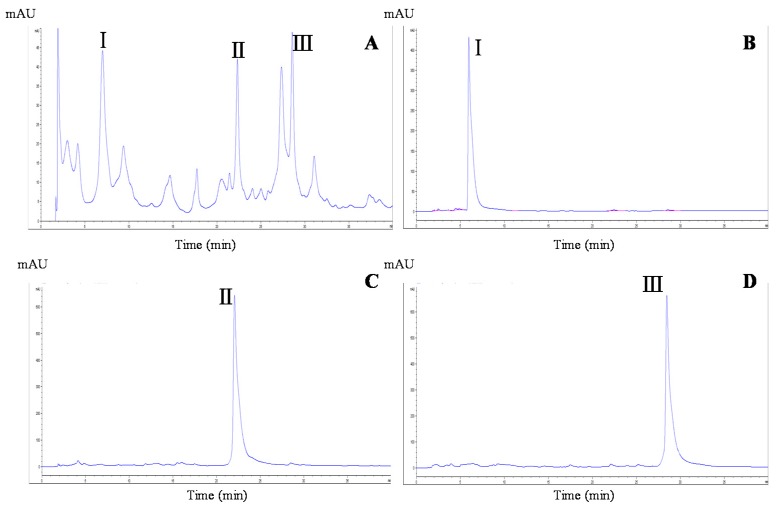
HPLC chromatograms of the MBE and HSCCC peak fractions. (**A**) MBE; (**B**) peak 1 in Figure 2; (**C**) peak 2 in Figure 2; (**D**) peak 3 in Figure 2.

In HSCCC-based separation, the selection of a suitable two-phase solvent system is the most vital process. The appropriate biphasic system can provide an ideal range of the partition coefficient (*K*) for the target compounds, whose value is usually considered in the range of 0.5 to 2.0 [15]. In this experiment, four different volume rations of a two-phase solvent system of *n*-hexane/ethyl acetate/methanol/water were tested to obtain the optimum composition. The resulting *K* values of the target compounds are summarized in Table 1. The *n*-hexane/ethyl acetate/methanol/water solvent system used at a ratio of 1:1:1:1 (v/v/v/v) yielded low *K* values for the three compounds. At a ratio of 1:5:1:2, the *K* values were suitable for the separation of compounds **1** (*K* value: 0.73) and **2** (1.03), but not compound **3** (3.42). However, when used at a ratio of 1:5:1:5, *n*-hexane/ethyl acetate/methanol/water yielded *K* values for all three compounds that allowed their separation. Therefore, the latter two-phase solvent system was adopted for further HSCCC separation. As shown in Figure 2B, ~240 mg of MBE were separated and purified in one step by HSCCC under the optimal separation conditions, and the three peaks were well resolved in a single run. The separation time was ~210 min for each run. The three compounds were eluted with good resolution and in the order of their increasing *K* values. Thus, three fractions were collected, with compound **1** (18.3 mg) obtained from peak 1, compound **2** (20.5 mg) obtained from peak **2**, and compound **3** (28.4 mg) obtained from peak 3. The purity of each of the three target compounds was >92% as determined by HPLC (Figure 3A–D).

### 2.3. Structural Identification of Compounds

The structure of the isolated compounds was identified by ESI-MS, ^1^H-NMR, and ^13^C NMR (Figure 1).

Compound **1**: colorless solid, C_16_H_18_O_9_; ESI-MS: *m/z* 377 [M + Na]^+^; ^1^H NMR (600 MHz, CD_3_OD) *δ*_H_: 7.55 (1H, d, *J* = 15.6 Hz, H-7′), 7.05 (1H, d, *J* = 2.0 Hz, H-2′), 6.96 (1H, dd, *J* = 7.8, 1.8 Hz, H-6′), 6.78 (1H, d, *J* = 7.8 Hz, H-5′), 6.26 (1H, d, *J* = 15.6 Hz, H-8′), 5.33 (1H, ddd, *J* = 9.0, 9.0, 4.5 Hz, H-5), 4.17 (1H, ddd, *J* = 5.0, 3.5, 3.5 Hz, H-3), 3.73 (1H, dd, *J* = 8.5, 3. 0 Hz, H-4), ~2.03–2.21 (4H, m, H-2, 6); ^13^C NMR (150 MHz, CD_3_OD) *δ*_C_: 177.3 (C-7), 168.9 (C-9′), 149.7 (C-4′), 147.3 (C-7′), 146.9 (C-3′), 127.9 (C-1′), 123.2 (C-6′), 116.7 (C-5′), 115.4 (C-2′, 8′), 76.3 (C-1), 73.6 (C-4), 72.1 (C-5), 71.4 (C-3), 38.9 (C-6), 38.3 (C-2). Comparing the above data with the literature data [16], compound **1** was identified as chlorogenic acid.

Compound **2**: yellow amorphous powder, C_21_H_20_O_12_; ESI-MS: *m/z* 487 [M + Na]^+^; ^1^H NMR (600 MHz, DMSO-*d*_6_) spectrum showed signals at *δ*_H_ 12.63 (1H, s, OH-5), 6.20 (1H, d, *J* = 2.4 Hz, H-6), 6.40 (1H, *J* = 2.4 Hz, H-8), 6.84 (1H, d, *J* = 9.0 Hz, H-5′), and 7.58 (2H, m, H-6′, 2′) ascribed to quercetin moiety. The anomeric proton signal of glucoside residue was observed at *δ*_H_ 5.46 (1H, d, *J* = 7.8 Hz); ^13^C NMR (150 MHz, DMSO-*d*_6_) exhibited the presence of 21 signals, including 15 carbon resonances for flavonoid aglycone at *δ*_C_ 177.5 (C-4), 164.1 (C-7), 161.2 (C-5), 156.3 (C-2), 156.2 (C-9), 148.5 (C-4′), 144.8 (C-3′), 133.5 (C-3), 121.6 (C-1′), 121.1 (C-6′), 116.2 (C-5′), 115.2 (C-2′), 103.0 (C-10), 98.7 (C-6), and 93.5 (C-8), and sugar moiety at *δ*_C_ 60.2, 69.9, 74.1, 76.5, 77.6, and 100.9. These ESI-MS, ^1^H, and ^13^C NMR data were similar to those in a previous report [17]. Therefore, compound **2** was identified as quercetin-3-*O*-*β*-d-glucopyranoside.

Compound **3**: yellow amorphous powder, C_22_H_22_O_12_; ESI-MS *m/z* 501 [M + H]^+^, ^1^H NMR (CD_3_OD, 600 MHz) spectrum displayed flavonoid proton signals at *δ*_H_ 7.92 (1H, d, *J* = 1.8 Hz, H-2′), 7.44 (1H, dd, *J* = 8.4, 1.8 Hz, H-6′), 6.87 (1H, d, *J* = 8.4 Hz, H-5′), 6.17 (1H, br s, H-8), and 5.97 (1H, br s, H-6), an anomeric proton at *δ*_H_ 5.57 (1H, d, *J* = 7.5 Hz, H-1″), and a methoxyl group at 3.82 (3H, s, 4′-OMe); ^13^C NMR (150 MHz, DMSO-*d*_6_) spectrum flavonoid carbon resonances at *δ*_C_ 177.5 (C-4), 164.1 (C-7), 161.2 (C-5), 156.3 (C-2), 156.2 (C-9), 149.4 (C-4′), 146.9 (C-3′), 133.0 (C-3), 122.0 (C-1′), 121.1 (C-6′), 115.2 (C-5′), 113.5 (C-2′), 104.0 (C-10), 98.7 (C-6), and 93.5 (C-8). Glucoside residue carbon signals were observed at *δ*_C_ 60.1, 69.8, 74.1, 76.5, 77.6, and 100.8. These data were in good agreement with the reported compound **3** [18], isorhamnetin-3-O-*β*-d-glucopyranoside.

### 2.4. Antioxidant Activities of the Target-Isolated Compounds

Phenolic compounds are widely distributed in plants, vegetables, and fruits, and have been recognized as powerful in vitro antioxidants due to their ability to scavenge free radicals via donated hydrogen atoms or electrons [19,20]. Since the total antioxidant capacity of a compound cannot be determined in a single assay, we used a combination of free-radical scavenging methods to evaluate the antioxidant potential of compounds **1**–**3**.

DPPH is a stable organic nitrogen radical that has been used extensively as a basic screening method to test the antioxidant abilities of natural resources according to their hydrogen-donating ability [21]. The assay was conducted in methanol, and the results are expressed as IC_50_ values (Table 2). In the case of the three isolated compounds, the activity of compound **2** against DPPH was the highest (IC_50_ of 9.64 μg/mL), followed by compound **3** (18.45 μg/mL) and compound **1** (36.51 μg/mL). The IC_50_ of ascorbic acid, a free-radical scavenger used as the reference control in this study, was 10.42 μg/mL. The free-radical scavenging capacity of flavonoids (compounds **2** and **3**) is dependent on the catechol moiety in ring B [22]. Compound **2** contains one more hydroxyl group than compound **3**, and thus has a greater ability to quench DPPH radicals. Compound **1** possesses only one hydroxycinnamic acid moiety, and is thus a weaker scavenger compared with compounds **2** and **3**.

Large quantities of superoxide radicals are generated in the body by a variety of metabolic and physiological processes [23]. Superoxide radicals are the precursors of singlet oxygen and hydroxyl radicals, both of which may be very harmful to cellular components [24]. Therefore, we examined the superoxide radical scavenging ability of the isolated compounds in a phenazine methosulfate–nicotinamide adenine dinucleotide superoxide-generating system using gallic acid as the control. As shown in Table 2, compound **2** exhibited significantly stronger superoxide radical scavenging ability than those of compounds **1** and **3**, based on the IC_50_ values. The structure–antioxidant relationship was similar to that determined using DPPH. The relative order of the three isolated compounds and gallic acid with respect to their superoxide radical scavenging capacities was compound **2** > gallic acid > compound **1** > compound **3**.

### 2.5. Cytoprotective Abilities and Inhibition of Lipid Peroxidation in H_2_O_2_-Treated RAW264.7 Cells

**Figure 4:**
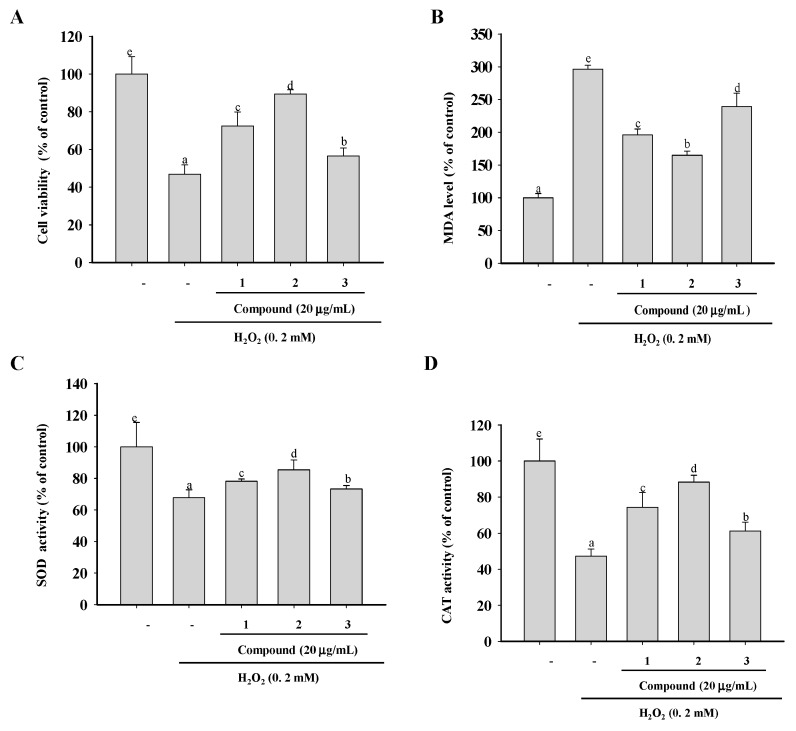
Cytoprotective activities of compounds **1**–**3**. (**A**) RAW 264.7 macrophages were exposed to the test compounds for 0.5 h and then to 200 μM of H_2_O_2_ for 6 h. Cell viability was measured using the 1-(4,5-dimethylthiazol-2-yl)-3,5-diphenylformazan (MTT) assay. (**B**) The malondialdehyde (MDA) content and activities of the antioxidant enzymes superoxide dismutase (SOD) (**C**) and catalase (CAT) (**D**) were determined using commercial kits according to the manufacturers’ instructions. The data are presented as means ± SD (n = 3). Values with the same superscript letters are not significantly different from each other at *p* < 0.05.

The in vitro chemical assays employed in this study do not reflect the physiological conditions of cells. H_2_O_2_, a form of reactive oxygen species (ROS), can penetrate cell membranes and cause oxidative cellular damage [25]. Its suppression may prevent H_2_O_2_-mediated oxidative stress in cells [26]. Therefore, we examined the ability of compounds **1** to **3** to scavenge free radicals in a cellular environment by assaying their protection against H_2_O_2_-induced oxidative damage in the macrophage cell line RAW264.7. An initial determination of the cytotoxic dose of H_2_O_2_ in RAW 264.7 cells showed moderate cell injury induced by 200 μM of H_2_O_2_. Therefore, RAW264.7 cells were first treated for 0.5 h with non-toxic concentrations (data not shown) of compounds **1**–**3**, and then for an additional 12 h with H_2_O_2_. As shown in Figure 4A, compared with the vehicle-treated group, there was a significant decrease in the viability of cells exposed to H_2_O_2_. However, in cells pretreated with the three compounds, viability was significantly higher than that in the vehicle group, and the highest values corresponded to compound **2**, followed by compound **1** and **3**.

The presence of unsaturated phospholipids in cell membranes render them vulnerable to free radicals, which initiate a chain reaction that results in the accumulation of end products such as malondialdehyde (MDA) and unsaturated aldehydes [27,28]. To evaluate the ability of compounds 1–3 to protect cells against macromolecular damage following H_2_O_2_ exposure, we followed the formation of MDA as a marker of oxidative damage. As shown in Figure 4B, treatment with H_2_O_2_ enhanced lipid peroxidation and MDA levels significantly (296.19%) compared with the control group, demonstrating that cellular polyunsaturated fatty acids underwent H_2_O_2_-mediated oxidative damage. However, the preincubation of the cells with compounds **1**–**3** significantly lowered MDA levels to 196.23%, 164.91%, and 239.31%, respectively. These results again demonstrate the greater potency of compound **2** in the scavenging of ROS and the inhibition of H_2_O_2_-induced lipid peroxidation.

Generally, cells respond to oxidative stress with adaptive changes designed to prevent cellular damage and increase survival [29]. Among the components of the cellular defense system against oxidative stress are the antioxidant enzymes superoxide dismutase (SOD) and catalase (CAT), which provide first-line cellular protection against excess amounts of free radicals [30,31]. The treatment of RAW264.7 cells with 200 μM of H_2_O_2_ for 12 h significantly decreased the activities of SOD and CAT by 67.76% and 42.29%, respectively, indicating impaired antioxidant defenses (Figure 4C,D). However, preincubation with compounds **1**–**3** significantly attenuated the H_2_O_2_-induced changes in SOD and CAT.

## 3. Materials and Methods

### 3.1. Material and Reagents

*M. bealei* leaves were purchased from Guizhou Province (China) in 2013, and the voucher specimen (MBL-2013) was deposited in the Jiangsu Collaborative Innovation Center Of Regional Modern Agriculture and Environmental Protection. The crude extract and each fraction were from *M. bealei* leaves were prepared as described previously [11]. Since the ethyl acetate fraction of *M. bealei* leaf extract (MBE) was more efficacious than the other fractions for scavenging free radicals, the MBE fraction was considered for ongoing research. 1,1-Diphenyl-2-picryl-hydrazyl (DPPH), phenazine methosulfate (PMS), nitro blue tetrazolium (NBT), β-nicotinamide adenine dinucleotide (NADH), and 1-(4,5-dimethylthiazol-2-yl)-3,5-diphenylformazan (MTT) were purchased from Sigma (St. Louis, MO, USA). Fetal bovine serum (FBS) was obtained from Corning (Mediatech, Manassas, VA, USA). The Roswell Park Memorial Institute (RPMI) 1640 medium was purchased from Gibco BRL (Life Technologies, China). Penicillin–streptomycin solution was acquired from Gibco BRL (Grand Island, NY, USA). Phosphate-buffered saline (PBS, pH 7.4) tablets were obtained from Amresco (Solon, OH, USA). Assay kits for catalase (CAT), malondialdehyde (MDA), and superoxide dismutase (SOD) were purchased from the Institute of Nanjing Jiancheng Bioengineering (Nanjing, China). The organic solvents used for fraction and HSCCC separation were purchased from Sinopharm Chemical Reagent Co., Ltd. (Shanghai, China). All the aqueous solutions were prepared with deionized water produced by Millipore Direct-Q3 Water Purification system (Bredford, MA, USA).

### 3.2. Apparatus

The preparative HSCCC instrument employed in this study was a Tauto TBE-300B (Shanghai, China). It was equipped with three polytetrafluoroethylene coil columns, a Tauto TBP-5002 constant-flow pump, a QuikSep UV-50 UV detector (H&E Factory, Beijing, China), and a DBS-100 mode fraction collector (Shanghai Huxi Instruments Factory, Shanghai, China). The Agilent 1260 system (Santa Clara, CA, USA) was employed for HPLC-UV analysis. ESI-MS spectra were obtained with an LTQ-Orbitrap XL spectrometer (Thermo Fisher Scientific, Bremen, Germany). ^1^H NMR and ^13^C NMR data were recorded using a Bruker Avance 600 NMR spectrometer (Fällanden, Switzerland).

### 3.3. Measurement of the Partition Coefficient (K)

The procedure used to determine *K* was as follows: approximately 5 mg of MBE was dropped into a test tube, and 2 mL of equilibrated two-phase solvent was added to the tube. The glass tube was shaken vigorously for 2 min to separate the upper and lower phases completely. Then, one milliliter of each phase was dried under N_2_ at 40 °C and re-dissolved in acetonitrile for HPLC. The *K* value for each target compound was calculated as the ratio of each peak in the upper and lower phases.

### 3.4. HSCCC Separation

A two-phase solvent system consisting of *n*-hexane/ethyl acetate/methanol/water (1:5:1:5, v/v/v/v) was selected for HSCCC separation. Each solvent was added into a separate funnel and shaken vigorously at room temperature. The two phases were separated and degassed by sonication before use. The coil column was first entirely filled with the upper phase by a constant flow pump; subsequently, the lower mobile phase was then pumped into the inlet of the column at a flow rate of 2 mL/min, after which the apparatus was rotated at a revolution speed of 900 rpm in tail–head elution mode. After a hydrodynamic equilibrium was established, an approximately 10-mL sample solution was injected into the injection valve. The peak fractions were manually collected according to the chromatogram with a UV detector at 254 nm and then evaporated under a vacuum rotary evaporator.

### 3.5. In Vitro Antioxidant Activity

#### 3.5.1. DPPH Free-Radical Scavenging Assay

The antioxidant potency of the different samples was determined based on the method [32]. Briefly, a methanol solution (100 μL of the sample at various concentrations) was mixed with 100 μL of methanol solution of DPPH (0.2 mM). After reacting for 30 min in the dark at room temperature, the optical density was read at 517 nm in a spectrophotometer. The concentrations of test samples at which there is a 50% fall in the absorbance of DPPH values (IC_50_) were calculated by a dose–response curve using Microsoft Office Excel 2010 (Microsoft Corp., Redmond, WA, USA).

#### 3.5.2. DPPH–HPLC Experiment

The DPPH–HPLC experiment was carried out according to the previous literature with slight modifications [33]. Briefly, a volume of 200 μL of 5 mg/mL MBE was mixed with DPPH solution (0.2 mM in acetonitrile). After passing through a 0.45-μm syringe filter, the reaction mixtures were analyzed by HPLC in comparison with a blank and control. The mixtures were separated and analyzed by a reversed-phase ZORBAX ODS (150 mm ×4.6 mm i.d., 5 μm, Milford, MA, USA) column. The mobile phase consisted of A (0.4% acetic acid in water) and B (acetonitrile), which was programmed as follows: 0–40 min, 10–25% B. The flow rate was 1 mL/min, while the ambient temperature was controlled at 30 °C, and 254 nm was selected as the detection wavelength.

#### 3.5.3. Superoxide Radical Scavenging Assay

The superoxide radical scavenging activity of different samples was measured by the reduction of NBT according to the previously reported method with minor modifications [34]. The reaction mixture consisted of 100 μL of each sample, 1 mL of 0.1 M phosphate buffer (pH 7.4), 100 μL of NBT (150 μM), 100 μL of NADH (468 μM), and 20 μL of PMS (60 μM). After 5 min of reaction at ambient temperature, the absorbance of the aqueous solution was determined at 560 nm.

#### 3.5.4. Cell Line and Cell Culture

The RAW264.7 mouse macrophage cell line was provided by the American Type Culture Collection (ATCC) (Manassas, VA, USA). RAW264.7 was plated into T-75 flasks and grown in RPMI 1640 medium containing 10% heat-inactivated FBS and 1% antibiotic solution (penicillin/streptomycin) at 37 °C in a humidified atmosphere of 95% air and 5% CO_2_ (95% air, 5% CO_2_).

#### 3.5.5. Cell Viability Assay

Cytotoxicity was investigated by MTT assay as previously described [35]. Prior to the experiment, cells were seeded into 96-well plates at a density of 1 × 10^5^ cells/well for 18 h. Then, the culture medium was replaced with fresh medium along with the test compounds at various concentrations for 1 h and exposed to 0.2 mM of H_2_O_2_ for an additional 12 h. Following this, the medium was carefully removed, and 10 µL of MTT solution (5 mg/mL in PBS) and 90 µL of FBS-free medium were added to each well and incubated at 37 °C for 4 h. The formazan crystals were solubilized in MTT stop buffer overnight. The absorbance was measured at 550 nm using a microplate multi-well reader (Tecan, Zurich, Switzerland). Cell viability was expressed as a percentage of control cells.

#### 3.5.6. Measurement of Lipid Peroxidation and CAT and SOD Activities

A total of 2 × 10^6^ cells were seeded in six-well plates for 16 h. Then, the culture medium was replaced with fresh medium and treated as described in Section 3.5.5. After treatment, cells were washed twice with ice-cold PBS and dissociated by cell lysis buffer. The supernatant was collected for measuring the MDA levels and activities CAT and SOD using assay kits according to the protocol of the manufacturer. The protein content was measured by with a MicroBCA protein assay kit (CWBIO, Beijing, China) using bovine serum albumin as the standard.

### 3.6. Data Analysis

All analyses were performed using SPSS 19.0 package (SPSS Inc., Chicago, IL, USA) for Windows 7. Values shown represent the mean ± standard derivation (SD). Differences among samples were compared using a Duncan’s multiple range test, and *p*-values less than 5% were considered to be statistically different.

## 4. Conclusions

Target-guided isolation way was set up to isolate three main antioxidants from *M. bealei* leaves using HSCCC. The described method also has broader applicability in the screening and preparation of other free radical scavengers present in crude plant extracts. The results obtained in this study clearly demonstrate the potent free-radical scavenging activities of compounds **1**–**3** and their ability to reduce oxidative stress in H_2_O_2-_treated RAW264.7 cells by decreasing intracellular MDA levels and increasing SOD and CAT activities. In all assays, a consistently similar trend was established with respect to the antioxidant activities of the three compounds, which were ranked as follows: compound **2** > compound **1** > compound **3**. The identification of these antioxidants should stimulate further research aimed at identifying other important molecules in *M. bealei* leaves and their applications in the food and pharmaceutical industries.

## Figures and Tables

**Table 1:** The partition coefficients (*K* values) of compounds **1**–**3** in the two-phase solvent systems of *n*-hexane/ethyl acetate/methanol/water as determined by HPLC.

No.	Ratio (v/v)	*K* values		
		I	II	III
1	1:1:1:1	0.09	0.23	0.32
2	1:2:1:2	0.21	0.45	0.57
3	1:5:1:2	0.73	1.03	3.42
4	1:5:1:5	0.65	1.21	1.86

**Table 2:** Free-radical scavenging activities of isolated compounds from *M. bealei* leaves.

Samples	DPPH (IC_50_, μg/mL)	•O_2_^−^ (IC_50_, μg/mL)
Compound **1**	18.45 ± 2.34 ^c^	134.15 ± 11.49 ^c^
Compound **2**	9.64 ± 0.52 ^a^	85.54 ± 5.32 ^a^
Compound **3**	36.51 ± 4.16 ^d^	206.86 ± 20.76 ^d^
Ascorbic acid	10.42 ± 0.72 ^b^	-
Gallic acid	-	96.85 ± 4.39 ^b^

Each value is the mean ± SD of triplicate measurements. Values with different letters differ significantly (*p* < 0.05).
